# Intraplacental choriocarcinoma and spontaneous fetomaternal hemorrhage: Uncovering diagnostic clues in a challenging case

**DOI:** 10.3389/fonc.2025.1600200

**Published:** 2025-09-01

**Authors:** Li Wang, Xuri Li, Dongfang Bai, Ran Du, Linna Lu, Yujie Li, Xiuyu Wang

**Affiliations:** ^1^ Department of Gynecology and Obstetrics, Liaocheng People’s Hospital, School of Medicine, Liaocheng University, Liaocheng, China; ^2^ Department of Gynecology and Obstetrics, The Second People’s Hospital of Liaocheng, Shandong University, Jinan, China; ^3^ Department of Gynecology Oncology, Shandong Provincial Key Medical and Health Laboratory of Liaocheng Infectious Disease Hospital, Liaocheng, China; ^4^ Department of Gynecology and Obstetrics Qingdao Hospital of Traditional Chinese Medicine , Qingdao, China; ^5^ School of Medicine, Liaocheng University, Liaocheng, China; ^6^ Department of Pathology, Liaocheng People’s Hospital, Liaocheng, China

**Keywords:** intraplacental choriocarcinoma, fetomaternal hemorrhage, pathology, clinical characteristics, outcomes

## Abstract

**Background:**

Intraplacental choriocarcinoma (IC) is an extremely rare malignancy of the placenta that is often associated with fetomaternal hemorrhage (FMH). This combination is challenging to diagnose antepartum and is associated with high perinatal mortality, predominantly due to severe fetal anemia.

**Methods:**

We report a case of IC associated with FMH and conducted a systematic review of cases published in the English and Chinese literature. Data from these cases were combined to evaluate clinical characteristics, diagnostic approaches, treatment options, and outcomes.

**Results:**

A 31-year-old woman presented with decreased fetal movements at 34 weeks and 2 days of gestation. Following an emergency cesarean section, the infant was found to be severely anemic and passed away shortly after delivery. The placental histopathology revealed IC. The patient received chemotherapy with EMA/CO and showed no evidence of metastasis or recurrence over 12 months of follow-up. The systematic review identified 35 cases of IC associated with FMH. Common diagnostic clues included decreased fetal movements, abnormal middle cerebral artery Doppler studies, and FMH. Despite the severe perinatal outcomes, maternal prognosis was generally favorable with chemotherapy in case of metastasis.

**Conclusion:**

IC with FMH is a rare but life-threatening condition requiring high clinical suspicion and prompt placental histopathology for diagnosis. Early recognition of fetal anemia and FMH is essential for timely intervention.

## Introduction

Gestational trophoblastic disease (GTD) encompasses a diverse spectrum of lesions characterized by the abnormal proliferation of trophoblastic tissue arising from the placenta (1). The pathogenesis of GTD is distinctive, as these maternal lesions derive from fetal rather than maternal tissue. Gestational choriocarcinoma represents a rare and aggressive trophoblastic malignancy composed of neoplastic syncytiotrophoblasts, intermediate trophoblasts, and cytotrophoblasts (2). Choriocarcinoma is a rare and highly aggressive malignant trophoblastic neoplasm, which may arise from either gestational or non-gestational origins.

Intraplacental choriocarcinoma (IC) is an extremely rare form of gestational trophoblastic neoplasia, with choriocarcinoma identifiable within the placenta at any stage of gestation. Initially described in 1949 by Douglas and Otto (3) as “chorionepithelioma”, the entity was later referred to as “incidental choriocarcinoma” by Driscoll in 1963 (4), following its identification in a term placental specimen. Despite its clinical and pathological significance, IC remains relatively obscure. Case studies at a center for gestational trophoblastic disease detected IC in 0.04% of gestational trophoblastic neoplasia (5). Although heightened awareness of placental pathology has led to increased recognition of IC, fewer than 100 cases have been reported globally to date. The true incidence of IC is likely underestimated, as only 30% to 35% of placentas undergo pathological examination at the time of delivery.

Spontaneous fetomaternal hemorrhage (FMH) is defined as the transfer of fetal blood into the maternal circulation in the absence of trauma or clinical/histopathological evidence of placental abruption. The frequency and volume of small, spontaneous bleeds increase with advancing gestational age, peaking at the time of delivery (6). The incidence of spontaneous massive FMH remains unknown due to its rarity, unpredictability, and the nonspecific or often absent nature of its signs and symptoms (7). Spontaneous massive FMH can occur at any time during the antepartum or intrapartum period. Fetal death may be the initial manifestation of a massive acute hemorrhage. Alternatively, hydrops, abnormal fetal heart rate (FHR) patterns, and/or reduced fetal movement may signal a massive but non-lethal acute FMH or chronic intermittent FMH, resulting in substantial cumulative blood loss over time. In some instances, massive FMH may present without any overt clinical signs or symptoms. The pathogenesis of spontaneous massive FMH remains poorly understood. Histopathological evaluation of the placenta is crucial, as it may provide valuable insight into the underlying etiology of the FMH. Although rare, massive FMH has been reported to be associated with intraplacental choriocarcinoma (8–13).

We present a case of coexisting IC and FMH from the Gynecology and Obstetrics Centre in Liaocheng City along with a systematic review of the current literature. This review aims to evaluate the clinical characteristics, diagnostic approaches, treatment strategies, and outcomes for both the mother and the infant. Based on this comprehensive analysis, we highlight the importance of early diagnosis and effective management of this rare and complex condition in clinical practice.

## Method

We reported the case and gathered information from the electronic records system while providing the patient with a 12-month follow-up.

The PubMed database was searched from 1984 onwards, with the search strategy [(choriocarcinoma) OR (intraplacental choriocarcinoma) OR (intraplacental choriocarcinoma) OR (Gestational Trophoblastic Disease) OR (Gestational Trophoblastic Neoplasia) OR (Gestational Trophoblastic Tumors) OR (Placental Trophoblastic tumor)] AND [(fetomaternal hemorrhage) OR (FMH) OR (fetomaternal transfusion)] in February 2024 (**Supplementary 1**). References cited in the identified publications were reviewed to identify additional relevant cases. Reports in both English and Chinese literature describing histologically confirmed intraplacental choriocarcinoma diagnosed at any stage of pregnancy and associated with fetomaternal hemorrhage were included in this literature review. The review was not registered in PROSPERO due to the limited number of reported cases available. An initial search yielded 40 articles, of which four irrelevant studies were excluded through evaluation of contents. Six non-English-language studies (four in French, one in Germany, and the other in Italian) and one case without histological confirmation were further excluded. Finally, 30 articles concerning 34 cases of histologically or clinically confirmed IC published between 1984 and 2024 were included in the review (**Supplementary 2**). Data from the literature cases were consolidated to evaluate the presenting features, diagnostic pathways, maternal and fetal outcomes, and the efficacy of treatments prior to and following diagnosis.

The studies involving humans were approved by the Ethics Committee of Liaocheng People’s Hospital (no. 2024037). The studies were conducted in accordance with the local legislation and institutional requirements. The participants provided their written informed consent to participate in this study.

## Results

### Case presentation

A 31-year-old woman, gravida 2 para 1 (with normal-term deliveries), presented to the obstetric assessment unit at 34 weeks and 2 days of gestation with decreased fetal movements. The ultrasound examination revealed elevated middle cerebral artery peak systolic velocity suggestive of fetal anemia. The CTG demonstrated the disappearance of fetal heart rate variation. Based on these findings, the fetus was considered at high risk for fetal distress, so an emergency cesarean section was performed, resulting in the birth of a female infant. The infant weighed 2,300 g and had Apgar scores of 3 at 1 min and at 5 min, respectively. She was found to be severely anemic (Hb 22 g/L, Hct 7.9%), and the maternal serum alpha-fetoprotein (AFP) was 3,000 ng/mL. No hemolysis was observed. The infant was transferred to the neonatal intensive care unit, and a blood transfusion was planned. However, the parents declined the further treatment, and the infant passed away 1 day after delivery. The final diagnosis was severe anemia secondary to FMH.

**Figure 1:**
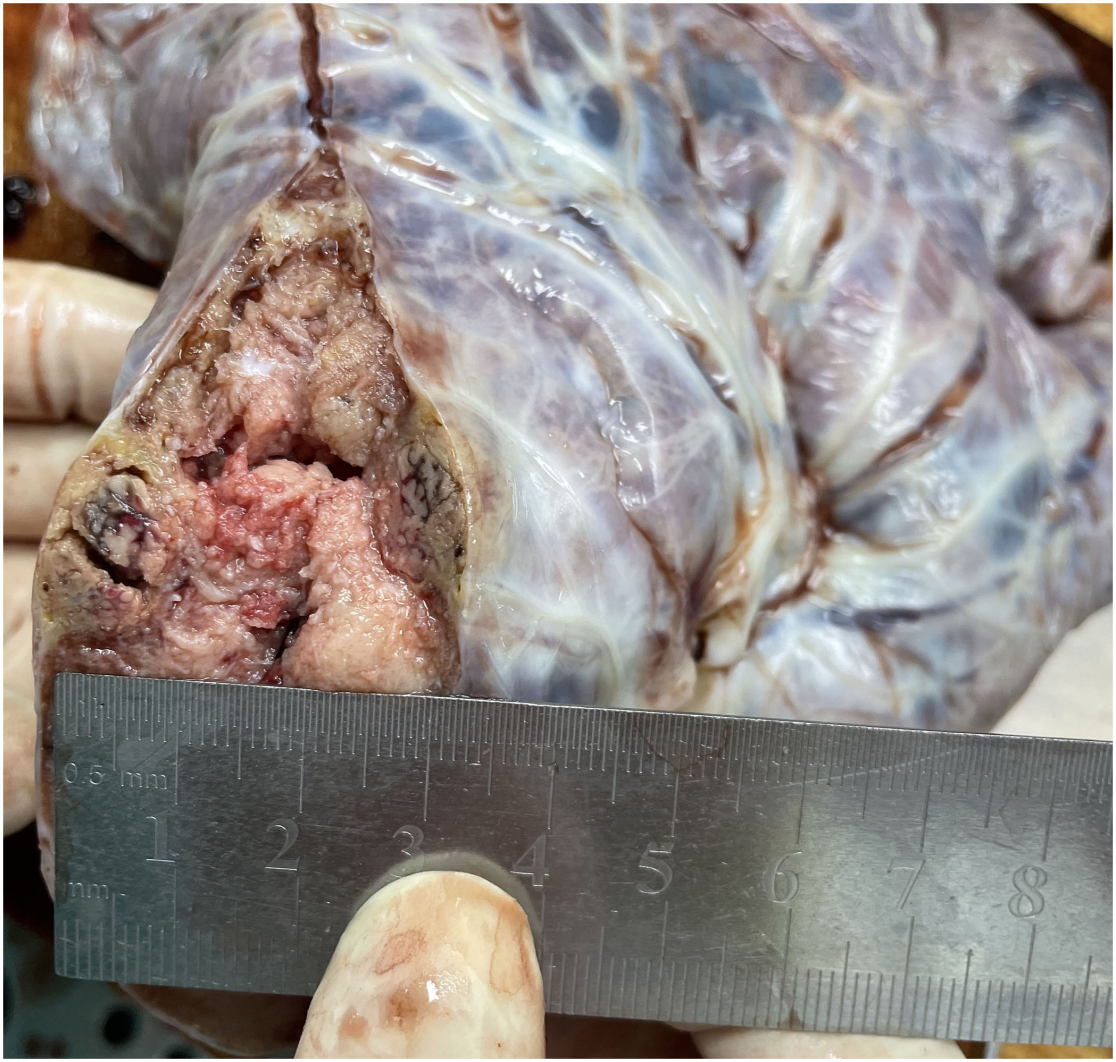
A yellow-gray area measuring 1.5 × 1.3 × 0.8 cm was observed on the cut surface near the fetal side, located 3 cm from the umbilical cord and indistinct from the surrounding tissue.

**Figure 2:**
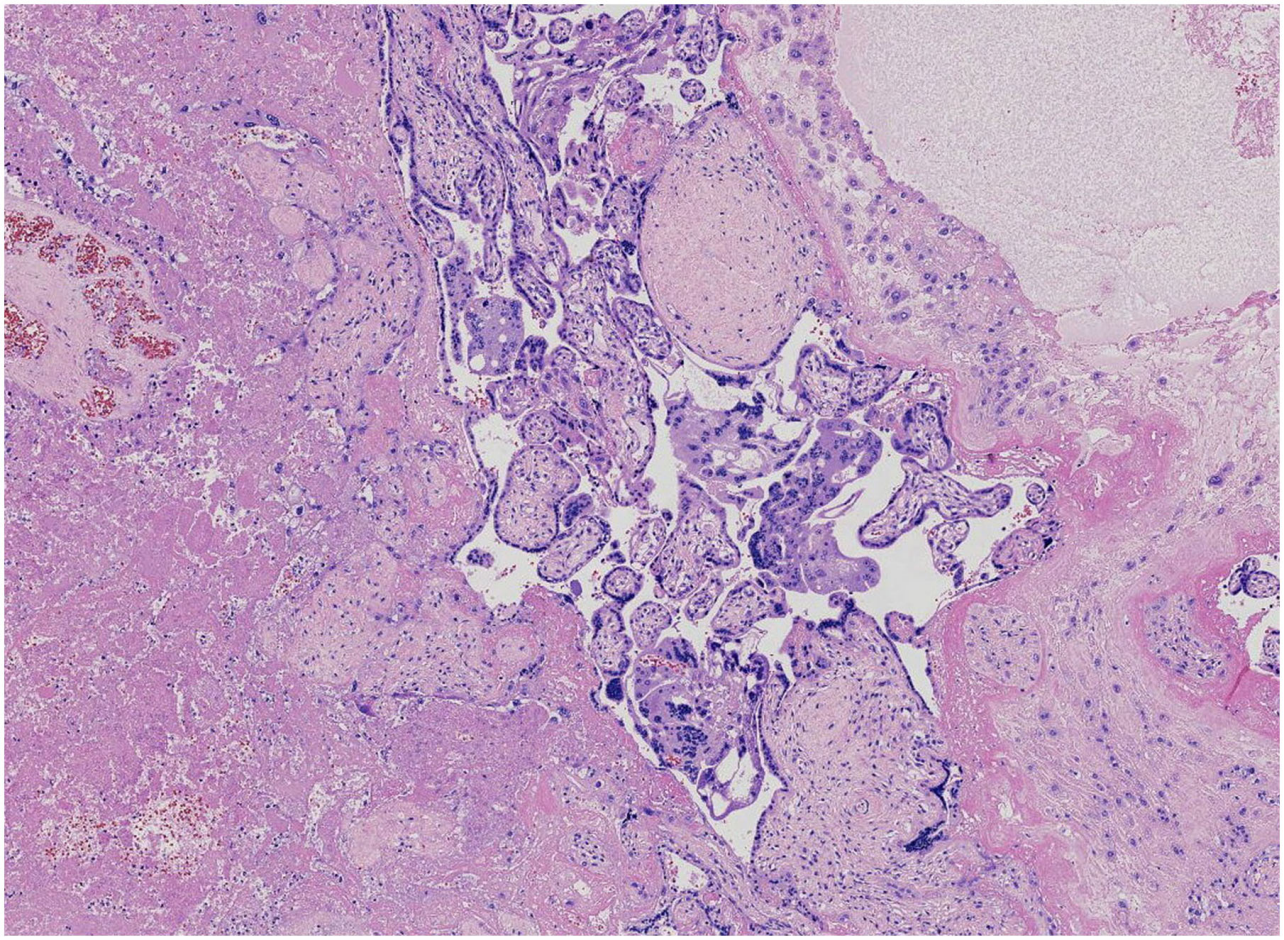
The residual tumor cells surrounding the necrotic lesions consist of syncytiotrophoblasts and mononuclear trophoblasts.

**Figure 3:**
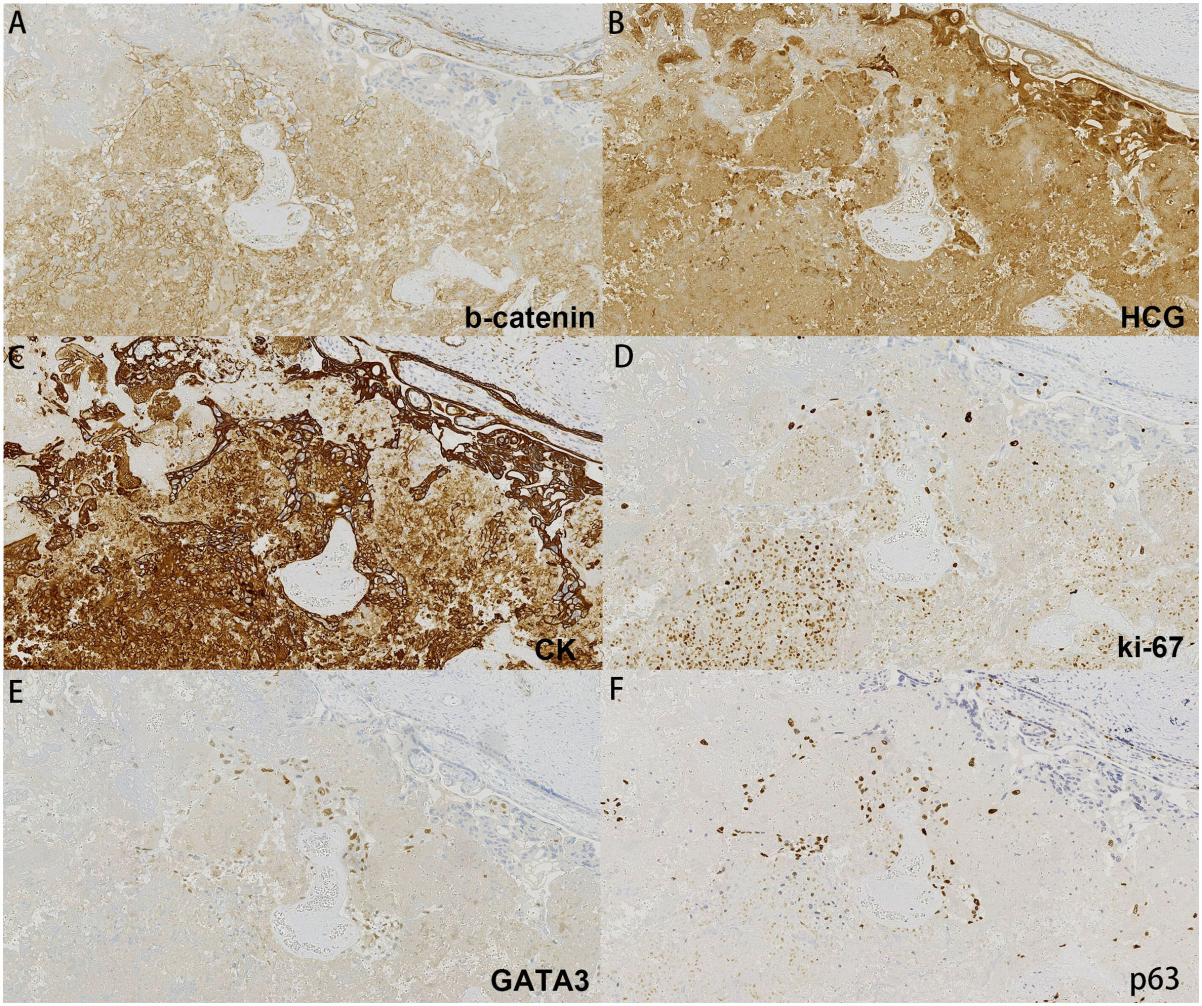
Immunohistochemistry of our case: **(A)** b-catenin, **(B)** HCG, **(C)** CK, **(D)** ki-67, **(E)** GATA3, **(F)** p63.

The placenta appeared immature, weighing 628 g, with dimensions of 18 × 18 × 5 cm. A yellow-gray area measuring 1.5 × 1.3 × 0.8 cm was observed on the cut surface near the fetal side, located 3 cm from the umbilical cord and indistinct from the surrounding tissue (**Figure 1**). The histological examination revealed a predominantly necrotic choriocarcinoma composed of atypical syncytiotrophoblasts and cytotrophoblasts (**Figure 2**). The immunohistochemical analysis demonstrated partial positivity for GATA3 and p63, diffuse positivity for cytokeratin, β-catenin, and β-HCG, and Ki-67 proliferation index of 50x% (**Figure 3**), confirming the diagnosis and distinguishing the tumor from other trophoblastic diseases. No evidence of choriocarcinoma was found in the other placental sections.

The postpartum maternal β-HCG level was 808.34 mIU/mL, and the computed tomography (CT) scans of the head, lungs, abdomen, and pelvis revealed no abnormalities. The patient received three courses of EMA/CO chemotherapy (etoposide, methotrexate, actinomycin D, cyclophosphamide, and vincristine). She was managed conservatively with serial β-HCG monitoring over 12 months, during which the levels remained consistently <0.5 mIU/mL.

### System review

A systematic review of the literature identified 30 articles describing 34 cases of confirmed IC associated with FMH (1, 8, 10–34). Reflecting the rarity of IC coexisting with FMH, only two of these reports (8%) involved multiple cases, suggesting potential data heterogeneity. The clinical characteristics, diagnostic approaches, treatments, and outcomes of all 35 patients, including the case reported here, are summarized below.

#### Maternal clinical characteristics

The median maternal age was 30 years (range, 17–47) with a median of two (range, 1–8) gestations, and median of no previous delivery (range, 0–7) prior to the presumed causative pregnancy. One of the women was 47 years old and gestating, while her husband was 71 years old (8). Among the 33 cases with gestational information available, one has a history of complete hydatidiform mole 2 years ago (12). One patient (3.13%) was diagnosed before 34 weeks (11) and 15 (46.88%) between 34 to 37 weeks, and the remaining 15 (46.88%) presented full-term pregnancy.

A total of 18 (54.55%) patients were asymptomatic, and the placental examination led to the diagnosis of IC because of fetal indications, where seven (21.21%) presented as obstetric complication including two (6.06%) preterm labor (17, 34), two (6.06%) preeclampsia (28), two (6.06%) premature rupture of membranes (18, 35), and one oligoamnios (36). Seven (21.21%) presented vaginal bleeding (12, 21, 23, 25, 31–33) and one (3.06%) has respiratory symptoms dyspnea (27) that was associated with disease-related symptoms. Where fetal presentation was concerned, 12 were asymptomatic, and 13 (38.24%) presented fetal distress and combination of several symptoms. All of the 13 cases showed abnormal cardiotocography such as non-reassuring fetal status pattern, preterminal pattern, or mild variable decelerations as well as occasional late decelerations while exhibiting reduced fetal movements; eight in 13 symptomatic patients showed intrauterine fetal death, and four (11.76%) cases were suspected of severe fetal anemia by ultrasound examination.

Excluding five cases without available data, histological confirmation of 18 (60%) IC was obtained 1 week post-delivery, with placental examination required shortly after delivery due to neonatal complications. In the other 12 cases, the histological diagnosis of IC was performed up to 7 months post-delivery (16). The histological examination of the placenta was delayed due to clinical backlog and completed when the fetal autopsy was conducted. In these women, 22 patients were free of metastatic disease or did not mention it. In 12 (35.29%) cases with metastases, pulmonary involvement was most common, affecting 11 patients (32.35%), followed by uterine (*n* = 8, 23.53%), brain, cerebral, and vaginal (2.94%, respectively) metastases.

Pretreatment serum hCG levels were available in 26 out of 29 patients. For patients with serum hCG measured in IU/L or mIU/mL (*n* = 26), the median level was 783.072 IU/L (range, 0.2–2,000,000 IU/L). The contribution of intraplacental choriocarcinoma-derived hCG to an overall raised level due to pregnancy is difficult to determine. Maternal blood type was described in 12 individuals, and half of these was type B rhesus positive.

Chemotherapy is the most popular treatment in 16/33 cases; 12 patients were expectant, and they were monitored by clinicians using serial blood tests until hCG was undetectable. One of the two women who underwent surgical treatment was given a combination of hysterectomy and chemotherapy, while the other had a hysterectomy performed. Of all the 34 cases with outcome available, 32 were kept healthy when they reported their cases of IC and FMH, while two women passed away because of cerebral hemorrhage (13) and respiratory failure induced by diffuse pulmonary fibrosis (11) (**Table 1**).

**Table 1:** Clinical characteristics of patients with intraplancentalchoriocarcinoma.

Characteristic		N	Median (range)/%
Age		34	30 (17-47)
Gestation*		33	2 (1-8)
Para		33	0 (0-7)
Preceding pregnancy		32	
	<34 weeks	1	3.13
	34-37 weeks	15	46.88
	>37 weeks	15	46.88
Maternal presentation		33	
	Asymptomatic	18	54.55
	Preterm labor	2	6.06
	Preeclampsia	2	6.06
	Vaginal bleeding	7	21.21
	Rupture of membranes	2	6.06
	Dyspnea	1	3.03
	Oligoamnios	1	3.03
Fetal presentation #		35	
	Asymptomatic	12	34.29
	Cardiotocography abnormal	13	37.14
	Reduced fetal movements	13	37.14
	IUFD	8	22.86
	Ultrasound examination suspected severe fetal anemia	4	11.43
Delivery		30	
	Vaginal	15	50
	Cesarean Section	15	50
Time to diagnosis after delivery		30	
	< 1 week	18	60
	1 week to 4 weeks	4	13.33
	4 week to 8 weeks	4	13.33
	> 8 weeks	4	13.33
Metastatic Site		35	
	No metastasis	23	65.71
	Metastasis	12	34.29
	Uterus	8	66.67
	Vagina	1	8.33
	Brain	1	8.33
	Pulmonary	11	91.67
	Other	1	8.33
Pretreatment HCG $		26	738.072 (0.2-2,000,000)
Blood Type		12	
	A Rh-	1	8.33
	A Rh+	6	50
	B Rh+	2	16.67
	O Rh+	2	16.67
	AB Rh+	1	8.33
Treatments &		33	
	Expectancy	12	36.36
	Chemotherapy	16	48.48
	Surgery	2	6.06
	No definitive treatment	4	12.12
Outcomes		34	
	Alive	32	94.12
	Death	2	5.88

* One woman has a history of complete hydatidiform mole two years ago.

# They may presented several symptoms at the same time.

$ 29 available, 2 case use urine HCG, 1 use dilution.

& 1 case surgery + chemotherapy.

#### Pathology

IC was confirmed by histopathological placental examination in all 35 patients included in this series. The placental weight was available in 15 cases; the median weight was 630 g (range, 270–1,300 g), three cases included a gross description of the placenta without reporting its weight, but noted that the placenta appeared large (23, 25, 31).

Macroscopically, the placenta was reported to look normal in 12 patients. Where described, the lesions in the other 13 cases resembled an infarct or thrombus, excluding a placenta that was damaged by ischemia or hypertension and those containing a single lesion as reported in 11 cases and multiple lesions in one case, with a median diameter of 20 mm (range, 10–60 mm). Eight cases reported the location details of the lesions on the placenta; 87.5% of them (7) were located at the margin, one case reported the presence of multiple lesions in different locations. Immunohistochemistry could help the diagnosis of IC. Here are nine cases which detailed their immunohistochemistry. The most commonly used was HCG (77.78%), followed by CK, Ki-67, p63, GATA-3, HPL, b-catenin, p57, AFP, and glypican-3 (**Table 2**).

**Table 2:** Indications for placental pathological examination.

Characteristic		N	Median (range)/%
Placental weight *		15	630 (270-1300)
Size of tumor (Largest radius)		19	20 (10-60)
Tumor location of the placenta		33	
	Not mentioned	25	75.76
	Description	8	24.24
	Margin	7	87.5
	Multiple areas	1	12.5
Immunohistochemistry		9	
	HCG	7	77.78
	CK	4	44.44
	Ki-67	4	44.44
	P63	3	44.44
	GATA-3	2	33.33
	HPL	2	22.22
	b-catenin	1	11.11
	P57	1	11.11
	AFP	1	11.11
	Glypican-3	1	11.11

* Here are 18 cases exhibited placental grossly description. 3 cases didn’t detail the placental weight but reported a large volume.

HCG, serum human chorionic gonadotropin; CK, cytokine;GATA-3, GATA binding protein 3; AFP, alpha-fetoprotein; HPL, human placental lactogen.

#### Fetal and infant cases

Of the cases with fetal and infants’ descriptions, 29 cases have birth information. The mean birth weight was 2,750 g (range, 1,290–3,884 g). The gender of the fetus at birth was female in 13 cases and male in 16 cases. In terms of the Apgar score, 20 cases with the 1 min score were available and 18 with 5 min. The median Apgar score of 1 and 5 min was 6.5 (0–9) and 7 (3–10), respectively. Of the 14 non-viable fetuses, IUFD occurred in eight cases (57.14%) without an otherwise complicated gestation, and the deaths were attributed to FHM. A total of 19 (54.29%) of 35 were diagnosed as anemia at birth clinically or diagnosed supported by HGB testing. Furthermore, 23 cases had data on hemoglobin level in these studies. The median HGB was 4.7 g/dL (1.2–7.8 g/dL). We also analyzed the blood type for fetuses and infants. In the five cases with blood type available, two cases reported type A Rh+ (22, 24), followed by type A Rh- (31), type B Rh+ (13), and type O Rh+ (27). In the 35 fetuses and infants, 16 were diagnosed by using Kleihauer–Betke test, and the others were diagnosed according to the clinical presentation with severe anemia, while four fetuses were diagnosed as anemia by ultrasound examination. A total of 20 infants received blood transfusion treatment, and three (10, 11, 26) of them died because of neonatal anemia and progressive multiple organ failure. Among the 24 infants with outcome data available, 19 were healthy until their reported study (**Table 3**).

**Table 3:** Clinical characteristics and outcomes of FMH infant and fetal.

Characteristic		N	Median (range)/%
Birth weight		29	2750 (1290-3884)
Gender		29	
	Female	13	44.83
	Male	16	55.17
Apgar 1min		20	6.5 (0-9)
Apgar 5min		18	7 (3-10)
Symptoms		35	
	anemia	19	54.29
	Neonatal hypoglycemia	1	2.86
	NA	15	42.86
HGB (g/dL)		23	4.7 (1.2-7.8)
Blood Type		5	
	A Rh-	1	20
	A Rh+	2	40
	B Rh+	1	20
	O Rh+	1	20
Diagnosis FMH		35	
	Kleihauer-Betke test	16	45.71
	Clinical presentation	19	54.29
Treatment		35	
	Blood Transfusion	20	57.14
	NA	15	42.86
Outcomes		32	
	IUFD	8	25
	Alive	19	59.38
	Died	5	15.63

HGB, hemoglobin; FMH, fetomaternal hemorrhage; IUFD, intrauterine fetal death.

## Discussion

Benson et al. (34) first described massive FMH as a complication of IC in 1962. Since then, several reports have documented the association between IC and FMH. Here we present a newly reported case along with the comprehensive review of this rare condition. Choriocarcinoma following a normal gestation is often comprised of biparental chromosomes identical to those of the fetus (37). Some choriocarcinomas arise within a term placenta and are referred to as IC. These are most commonly biparental in origin and do not necessarily follow a molar pregnancy (38). Choriocarcinoma may be either androgenetic or biparental, with the latter frequently associated with IC.

The incidence of IC is estimated to be approximately one in 50,000 pregnancies. However, as microscopic examination of the placenta is not routinely performed and is typically reserved for complicated pregnancies, its association with gestational events and adverse pregnancy outcomes is heavily influenced by ascertainment bias. IC is believed to be a potential source of metastatic disease following term pregnancies (39), and most cases of neonatal choriocarcinoma are attributed to metastatic spread from an intraplacental choriocarcinoma (5). Despite these findings, the true incidence of IC is likely underestimated, as placental histopathology is rarely conducted in all complicated pregnancies involving FMH or fetal distress. Furthermore, approximately half of IC cases are asymptomatic, and most pregnancies proceed uneventfully. Given that macroscopic placental examination alone has low sensitivity, it is probable that many cases remain undiagnosed, suggesting that the actual incidence may be higher than currently estimated.

Early predictors of FMH remain largely unidentified, with no cause determined in over 80% of cases (40). The placenta, often implicated in FMH (39), lies at the critical interface between maternal and fetal vascular systems, facilitating nutrient and waste exchange essential for sustaining fetal life *in utero*. Traditionally regarded as a fetal-derived, transient, and often overlooked organ (41), the placenta serves as a temporary structure supporting fetal growth and development (42). The placenta uniquely hosts cells from two genetically distinct individuals in close contact (43), not only facilitating nutrient exchange but also creating potential immunological challenges (44). In humans, the hemochorial placental structure and maternal–fetal villus interdigitation optimize fetal nutrition during long gestation but allow a direct interaction between embryonic trophoblasts and maternal blood. While generally immunologically tolerant, the maternal–fetal interface permits limited cellular trafficking (45, 46), including the passage of small amounts of fetal red blood cells (RBCs) into maternal circulation in nearly all pregnancies (47). FMH refers to the transfer of fetal blood from disrupted placental villi into the maternal intervillous space (48). Although volumes over 150 mL are considered massive FMH (49), the true incidence remains unclear due to its rarity and nonspecific symptoms (7). Physiological bidirectional RBC transfer is normal, but pathological disruption of the fetomaternal interface, often involving trophoblastic injury driven by inflammatory or mechanical factors (6, 50–56), underlies FMH (57). Despite ongoing research, the precise mechanisms of spontaneous massive FMH remain poorly understood. Two tests are available to estimate the volume of FMH: the Kleihauer–Betke acid elution assay and flow cytometry (58). Both assays are based on the identification of hemoglobin F, the predominant fetal hemoglobin. However, up to 1% of adult hemoglobin is hemoglobin F. However, these tests were not performed in our case due to the emergency nature of the clinical presentation at the time.

Histologic studies of placentas have shown that parenchymal and retroplacental hemorrhages, intervillous thrombi, and infarctions significantly increase the likelihood of fetal cells entering the maternal circulation, with the extent of these abnormalities correlating to the volume of hemorrhage (56). Despite these findings, many questions remain regarding the origins of these lesions and the factors influencing the frequency and magnitude of bleeding episodes. A histopathologic examination of the placenta is crucial as it may provide insights into the underlying cause of FMH. Although rare, massive FMH has been reported in association with intraplacental choriocarcinoma (8–13).

FMH can occur at any point during the antepartum or intrapartum period, and massive FMH cases may present without any overt signs or symptoms. Decreased or absence of fetal movement is the most frequent presenting symptom of massive FMH, while unexplained neonatal anemia (59, 60) is another important indicator. Massive intrapartum FMH leading to neonatal anemia has been reported in approximately one in 9,000 deliveries (61). Additionally, unexpected fetal demise or stillbirth is a critical diagnostic clue, with up to 15% of fetal deaths associated with massive FMH (61, 62). A histopathological examination of the placenta is essential as it may reveal the underlying cause of FMH. Although rare, massive FMH has been linked to intraplacental choriocarcinoma.

Diagnosis before delivery is critical, particularly in cases where decreased fetal movements are reported—a common symptom in almost all cases of FMH. Fetal cardiotocography (CTG) often reveals decreased variability, decelerations, and late decelerations, all of which suggest potential fetal compromise. When a suspected case of severe fetal anemia is identified based on CTG findings, ultrasonography is recommended for further evaluation. In most cases, ultrasound images of the umbilical artery and middle cerebral artery (MCA) show a normal flow pattern. However, the peak systolic velocity in the MCA (MCA-PSV) is markedly elevated—for instance, one report noted an MCA-PSV of 123 cm/s, significantly exceeding the normal range (1.5 multiples of the median = 82 cm/s). The MCA is a well-studied vessel, and ultrasound assessments of its PSV are routinely used to monitor fetuses at risk of placental compromise or fetal anemia (63). MCA-PSV has proven to be a reliable tool to predict and manage fetal anemia (15, 64, 65). While MCA abnormalities are not specific, they are invaluable in enhancing fetal surveillance, particularly when anemia is suspected (66).

When choriocarcinoma metastasizes, the most commonly affected sites include the lungs, brain, liver, pelvis, vagina, spleen, intestine, and kidneys. Among the 12 cases (35.29%) with documented metastases in this review, pulmonary involvement was the most prevalent, as observed in 32% of patients, followed by uterine, brain, cerebral, and vaginal metastases. Importantly, chemotherapy proved effective in cases of maternal metastasis, yielding favorable prognoses for these patients. Pathological examinations of the placenta were completed within 1 week in 18 cases, suggesting that IC has not yet garnered adequate attention for routine placental pathology evaluation among obstetric specialists. This underscores the necessity of raising awareness and providing clear diagnostic guidance to improve the recognition of IC.

We propose several key diagnostic clues that could aid in the early identification of IC, including decreased fetal movements, abnormalities in MCA, and the presence of FMH. These indicators should prompt obstetricians to consider placental pathology as part of their diagnostic workflow. Histologically, choriocarcinoma is characterized by sheets of anaplastic cytotrophoblasts and syncytiotrophoblasts in the absence of chorionic villi. Intermediate-appearing trophoblasts may also be present, but the biphasic pattern of clearly malignant mononuclear cytotrophoblasts and multinuclear syncytiotrophoblasts is pathognomonic of choriocarcinoma. Common histologic findings include extensive necrosis, hemorrhage, and vascular invasion. Immunohistochemical markers play a crucial role in confirming the diagnosis, with strong staining for human chorionic gonadotropin (hCG), inhibin, and cytokeratin in all trophoblast cells. Ki-67 is diffusely expressed in approximately 50% of the cells (67), reflecting the high proliferative activity of the tumor. Molecular markers commonly expressed by epithelioid trophoblastic tumor (ETT) through immunohistochemistry include pancytokeratin, epithelial membrane antigen, cytokeratin 18, inhibin-a, hCG, human placental lactogen (hPL), placental alkaline phosphatase (PLAP), and Mel-CAM (CD146). P63 is expressed in ETT, placental site nodules, and choriocarcinoma, but not in placental site trophoblastic tumor (PSTT) (68). PLAP staining is typically strong, while hPL, inhibin, and Mel-CAM staining tend to be weaker and more focal. It is important to note that ETT can sometimes be misdiagnosed as squamous cell carcinoma due to its involvement of the lower uterine segment or endocervix, its epithelioid histologic appearance, and its expression of p63 and cytokeratins. Therefore, a thorough pathological evaluation is essential to distinguish between these entities.

A recent review by Stabile et al. (19) analyzed 19 cases of IC complicated by FMH, providing valuable insights into maternal and fetal outcomes. Our larger case series, encompassing 35 patients including the present case, demonstrates concordant findings with their review. Both analyses report a similarly high perinatal mortality rate and underscore the severe fetal risk associated with this condition. Maternal metastases were observed in roughly one-third to 40% of cases, with pulmonary involvement being the most common site. Notably, chemotherapy was effective in most metastatic maternal cases, resulting in favorable outcomes and low maternal mortality. Our series reported no maternal deaths, whereas Stabile et al. documented a 5.5% maternal mortality rate. Prenatal diagnosis remains challenging due to the nonspecific clinical presentation. Both studies highlight decreased fetal movements, abnormal middle cerebral artery Doppler findings, and the presence of FMH as key diagnostic clues. These findings emphasize the necessity of heightened clinical suspicion and prompt placental histopathological examination in cases of unexplained fetal distress or FMH. While tumor size varied among cases, no clear correlation with fetal or maternal outcomes was identified.

The consistency between our findings and those of Stabile et al. reinforces the importance of multidisciplinary awareness to facilitate early detection and intervention. Differences in reported maternal mortality may reflect variations in sample size, case selection, and management protocols. Overall, these data advocate for routine placental pathology examination in complicated pregnancies to improve diagnostic accuracy and guide timely treatment, thereby enhancing maternal prognosis and informing fetal risk stratification.

This study is limited by the rarity of IC associated with FMH, with most data derived from case reports and small series, which may introduce publication bias favoring unusual or severe cases. Despite a comprehensive search, some relevant cases may have been missed due to incomplete indexing, language restrictions, or unpublished data. Additionally, heterogeneity in clinical presentation, diagnostic methods, and reporting reduce the ability to perform a detailed comparative analysis. These limitations restrict the generalizability of findings and underscore the need for larger, prospective studies to better understand and manage this rare condition.

## Conclusions

Intraplacental choriocarcinoma associated with fetomaternal hemorrhage is a rare but life-threatening condition, often presenting with severe fetal anemia and high perinatal mortality. Diagnosis is frequently delayed due to the absence of specific maternal symptoms or prenatal imaging features, and IC is typically identified postpartum following unexplained fetal distress, anemia, or stillbirth. When coexisting with massive FMH, IC may be misdiagnosed as more common obstetric complications such as placental abruption or alloimmunization. Our case highlights the importance of considering IC in the differential diagnosis of unexplained fetal anemia and underscores the critical role of placental pathological examination in all FMH cases. Early recognition, interdisciplinary vigilance, and β-hCG surveillance may enhance detection and improve clinical outcomes.

## Data Availability

The original contributions presented in the study are included in the article/**Supplementary Material**. Further inquiries can be directed to the corresponding author.

## References

[1] Jiao L Ghorani E Sebire NJ Seckl MJ. Intraplacental choriocarcinoma: Systematic review and management guidance. Gynecologic Oncol Jun. (2016) 141:624–31. doi: 10.1016/j.ygyno.2016.03.026, PMID: 27020699

[2] McCluggage WG Singh N Gilks CB. Key changes to the World Health Organization (WHO) classification of female genital tumours introduced in the 5th edition (2020). Histopathology. Apr. (2022) 80:762–78. doi: 10.1111/his.14609, PMID: 34996131

[3] Douglas GF Otts OM Jr. Chorionepithelioma associated with normal pregnancy. Am J obstetrics gynecology. (1949) 57:401–4. doi: 10.1016/0002-9378(49)90450-0, PMID: 18107645

[4] SG DRISCOLL. Choriocarcinoma an “incidental finding” within a term placenta. Obstetrics Gynecology. (1963) 21:96–101.

[5] Sebire NJ Lindsay I Fisher RA Seckl MJ. Intraplacental choriocarcinoma: experience from a tertiary referral center and relationship with infantile choriocarcinoma. Fetal Pediatr pathology. Jan-Feb. (2005) 24:21–9. doi: 10.1080/15227950590961180, PMID: 16175749

[6] Bowman JM Pollock JM Penston LE. Fetomaternal transplacental hemorrhage during pregnancy and after delivery. Vox sanguinis. (1986) 51:117–21. doi: 10.1111/j.1423-0410.1986.tb00226.x, PMID: 3095989

[7] Stefanovic V. Fetomaternal hemorrhage complicated pregnancy: risks, identification, and management. Curr Opin obstetrics gynecology. Apr. (2016) 28:86–94. doi: 10.1097/gco.0000000000000248, PMID: 26866844

[8] Henningsen AK Maroun LL Havsteen H Svare J. Massive fetomaternal hemorrhage caused by an intraplacental choriocarcinoma: a case report. Case Rep Med. (2010) 2010:767218. doi: 10.1155/2010/767218, PMID: 20204132 PMC2831481

[9] Ryu N Ogawa M Matsui H Usui H Shozu M. The clinical characteristics and early detection of postpartum choriocarcinoma. Int J gynecological cancer: Off J Int Gynecological Cancer Society. Jun. (2015) 25:926–30. doi: 10.1097/igc.0000000000000184, PMID: 24987912

[10] Aso K Tsukimori K Yumoto Y Hojo S Fukushima K Koga T. Prenatal findings in a case of massive fetomaternal hemorrhage associated with intraplacental choriocarcinoma. Fetal diagnosis Ther. (2009) 25:158–62. doi: 10.1159/000209201, PMID: 19293586

[11] Santamaria M Benirschke K Carpenter PM Baldwin VJ Pritchard JA. Transplacental hemorrhage associated with placental neoplasms. Pediatr pathology. (1987) 7:601–15. doi: 10.3109/15513818709161424, PMID: 3449817

[12] Nagel HT Vandenbussche FP Smit VT Wasser MN Peters AA. Intraplacental choriocarcinoma as an unexpected cause of intrauterine death at term. Int J gynecological cancer: Off J Int Gynecological Cancer Society. (2007) 17:1337–9. doi: 10.1111/j.1525-1438.2007.00987.x, PMID: 17511805

[13] She Q Cheng Z El-Chaar D Luo F Guo X Wen SW. Intraplacental choriocarcinoma coexisting with fetomaternal hemorrhage: Case report, chemotherapy management, and literature review. Med Apr. (2018) 97:e9977. doi: 10.1097/md.0000000000009977, PMID: 29620671 PMC5902268

[14] Hookins B Vatsayan A. Intraplacental choriocarcinoma and fetomaternal haemorrhage and maternal disseminated intravascular coagulopathy in a term pregnancy: A case report. Case Rep women's Health Jul. (2020) 27:e00216. doi: 10.1016/j.crwh.2020.e00216, PMID: 32420044 PMC7218156

[15] Mari G Deter RL Carpenter RL Rahman F Zimmerman R Moise KJ. Noninvasive diagnosis by Doppler ultrasonography of fetal anemia due to maternal red-cell alloimmunization. Collaborative Group for Doppler Assessment of the Blood Velocity in Anemic Fetuses. New Engl J Med Jan 6. (2000) 342:9–14. doi: 10.1056/nejm200001063420102, PMID: 10620643

[16] Monteiro S Burling M Doyle H. Late diagnosis of intraplacental choriocarcinoma co-existing with fetomaternal haemorrhage causing fetal demise: A case report. Case Rep women's Health Jul. (2021) 31:e00341. doi: 10.1016/j.crwh.2021.e00341, PMID: 34345596 PMC8319208

[17] Schepisi C Dunsmuir P Lipsett J Oehler MK. Intraplacental choriocarcinoma in twin pregnancy causing fetomaternal haemorrhage and single twin demise: case report. Case Rep Oncol Jan-Dec. (2023) 16:151–6. doi: 10.1159/000529736, PMID: 36935936 PMC10018420

[18] Simões M Vale G Lacerda C Pais P Pignatelli D. Fetomaternal hemorrhage: a clue to intraplacental choriocarcinoma and neonatal Malignancy. J maternal-fetal neonatal medicine: Off J Eur Assoc Perinatal Medicine Fed Asia Oceania Perinatal Societies Int Soc Perinatal Obstet. Dec. (2022) 35:6615–7. doi: 10.1080/14767058.2021.1918665, PMID: 33944655

[19] Stabile G Gentile RM Carlucci S Stampalija T Biffi S Oletto G. Maternal and fetal outcomes of intraplacental choriocarcinoma complicated by fetomaternal hemorrhage: a systematic review. J maternal-fetal neonatal medicine: Off J Eur Assoc Perinatal Medicine Fed Asia Oceania Perinatal Societies Int Soc Perinatal Obstet. Dec. (2023) 36:2285238. doi: 10.1080/14767058.2023.2285238, PMID: 38010764

[20] Ishiguro T Suda K Enomoto T. Biochemical analysis of intraplacental choriocarcinoma and fetomaternal transfusion. J obstetrics gynaecology Res Mar. (2017) 43:587–91. doi: 10.1111/jog.13232, PMID: 28168818

[21] Peng HH Ng ZP Tang YH Chua AA Huang KG. Term pregnancy with choriocarcinoma presenting as severe fetal anemia and postpartum hemorrhage. Taiwanese J obstetrics gynecology. Jun. (2016) 55:430–3. doi: 10.1016/j.tjog.2016.04.021, PMID: 27343330

[22] Takahashi H Matsuda H Mizumoto Y Furuya K. Intraplacental choriocarcinoma with fetomaternal hemorrhage: a case study and literature review. J perinatal Med. (2008) 36:178–81. doi: 10.1515/jpm.2008.021, PMID: 18331209

[23] Datta T Banerjee AK Adeyemo AK McCullough WL Duncan A. Intraplacental choriocarcinoma associated with pregnancy continuum: a diagnostic dilemma. J obstetrics gynaecology: J Institute Obstetrics Gynaecology. Oct. (2007) 27:731–2. doi: 10.1080/01443610701691965, PMID: 17999307

[24] Koike Y Wakamatsu H Kuroki Y Isozaki A Ishii S Fujitsuka S. Fetomaternal hemorrhage caused by intraplacental choriocarcinoma: a case report and review of literature in Japan. Am J perinatology. Jan. (2006) 23:49–52. doi: 10.1055/s-2005-918893, PMID: 16450273

[25] Lam CM Wong SF Lee KW Ho LC Yu VS. Massive feto-maternal hemorrhage: an early presentation of women with gestational choriocarcinoma. Acta obstetricia gynecologica Scandinavica. Jun. (2002) 81:573–6. doi: 10.1034/j.1600-0412.2002.810620.x, PMID: 12047317

[26] Chou HC Chen RL Yau KI Huang SF Ni YH Tang JR. Infantile choriocarcinoma with idiopathic massive fetomaternal hemorrhage. Med Pediatr Oncol Mar. (2002) 38:203–4. doi: 10.1002/mpo.1311, PMID: 11836724

[27] Lee KW Ho LC. A case of massive fetomaternal haemorrhage at term associated with choriocarcinoma. Aust New Z J obstetrics gynaecology. May. (1999) 39:274–6. doi: 10.1111/j.1479-828x.1999.tb03394.x, PMID: 10755801

[28] Suh YK. Placental pathology casebook. Choriocarcinoma in *situ* of placenta associated with transplacental hemorrhage. J perinatology: Off J California Perinatal Assoc Mar. (1999) 19:153–4. doi: 10.1038/sj.jp.7200103, PMID: 10642980

[29] Duleba AJ Miller D Taylor G Effer S. Expectant management of choriocarcinoma limited to placenta. Gynecologic Oncol Mar. (1992) 44:277–80. doi: 10.1016/0090-8258(92)90057-p, PMID: 1541441

[30] Tsukamoto N Matsumura M Matsukuma K Kamura T Baba K. Choriocarcinoma in mother and fetus. Gynecologic Oncol May. (1986) 24:113–9. doi: 10.1016/0090-8258(86)90014-4, PMID: 3699573

[31] Feldman K. Choriocarcinoma with neonatal anemia. New Engl J Med Apr 14. (1977) 296:880. doi: 10.1056/nejm197704142961516, PMID: 584929

[32] Kruseman AC van Lent M Blom AH Lauw GP. Choriocarcinoma in mother and child, identified by immunoenzyme histochemistry. Am J Clin pathology. Mar. (1977) 67:279–83. doi: 10.1093/ajcp/67.3.279, PMID: 320862

[33] Blackburn GK. Letter: Massive fetomaternal hemorrhage due to choriocarcinoma of the uterus. J pediatrics. Oct. (1976) 89:680–1. doi: 10.1016/s0022-3476(76)80420-9, PMID: 957021

[34] Benson PF Goldsmith KL Rankin GL. Massive foetal haemorrhage into maternal circulation as a complication of choriocarcinoma. Br Med J Mar 24. (1962) 1:841–2. doi: 10.1136/bmj.1.5281.841, PMID: 13867353 PMC1958028

[35] Juan H Xian L Li Z. A rare case report of placental choriocarcinoma during pregnancy complicated with massive fetal maternal blood transfusion. Electronic J Pract Gynecological Endocrinology. (2021) 8:87–9. doi: 10.3969/j.issn.2095-8803.2021.26.029

[36] Fuping G Jin W Ping M. Intraplacental choriocarcinoma: a case report. Chin J Diagn Pathology. (2020) 27:110+145. doi: 10.3969/j.issn.1007-8096.2020.02.013

[37] Gompel C SS. The placenta. In: Pathology in obstetrics and gynecology, vol. 448. Lippincott, Philadelphia (1994).

[38] Savage J Adams E Veras E Murphy KM Ronnett BM. Choriocarcinoma in women: analysis of a case series with genotyping. Am J Surg pathology. Dec. (2017) 41:1593–606. doi: 10.1097/pas.0000000000000937, PMID: 28877059

[39] Seckl MJ Sebire NJ Berkowitz RS. Gestational trophoblastic disease. Lancet (London England). Aug 28. (2010) 376:717–29. doi: 10.1016/s0140-6736(10)60280-2, PMID: 20673583

[40] Stroustrup A Plafkin C Savitz DA. Impact of physician awareness on diagnosis of fetomaternal hemorrhage. Neonatology. (2014) 105:250–5. doi: 10.1159/000357797, PMID: 24526231 PMC4012333

[41] Maltepe E Fisher SJ. Placenta: the forgotten organ. Annu Rev Cell Dev Biol. (2015) 31:523–52. doi: 10.1146/annurev-cellbio-100814-125620, PMID: 26443191

[42] Jaremek A Shaha S Jeyarajah MJ Jaju Bhattad G Chowdhury D Riddell M. Genome-wide analysis of hypoxia-inducible factor binding reveals targets implicated in impaired human placental syncytiotrophoblast formation under low oxygen. Am J pathology. Jul. (2023) 193:846–65. doi: 10.1016/j.ajpath.2023.03.006, PMID: 37028593

[43] Archibald JD Rose KD. Womb with a view: The rise of placentals. (2005) Cham, Switzerland: Springer International Publishing AG.

[44] Wildman DE Chen C Erez O Grossman LI Goodman M Romero R. Evolution of the mammalian placenta revealed by phylogenetic analysis. Proc Natl Acad Sci United States America. (2006) 103:3203–8. doi: 10.1073/pnas.0511344103, PMID: 16492730 PMC1413940

[45] Maloney S Smith A Furst DE Myerson D Rupert K Evans PC. Microchimerism of maternal origin persists into adult life. J Clin Invest. (1999) 104:41–7. doi: 10.1172/jci6611, PMID: 10393697 PMC408407

[46] Jonsson AM Uzunel M Götherström C Papadogiannakis N Westgren M. Maternal microchimerism in human fetal tissues. Am J obstetrics gynecology. Mar. (2008) 198:325. doi: 10.1016/j.ajog.2007.09.047, PMID: 18191801

[47] Medearis AL Hensleigh PA Parks DR Herzenberg LA. Detection of fetal erythrocytes in maternal blood post partum with the fluorescence-activated cell sorter. Am J obstetrics gynecology. (1984) 148:290–5. doi: 10.1016/s0002-9378(84)80070-8, PMID: 6421161

[48] Boller MJ Moore GS Hung YY Ritterman Weintraub ML Schauer GM. Fetomaternal hemorrhage: evaluation of recurrence within a large integrated healthcare system. Am J obstetrics gynecology. (2021) 225:540. doi: 10.1016/j.ajog.2021.04.257, PMID: 33961809

[49] de Almeida V Bowman JM. Massive fetomaternal hemorrhage: Manitoba experience. Obstet Gynecol. (1994) 83:323–8., PMID: 8127519

[50] Bianchi DW Romero R. Biological implications of bi-directional fetomaternal cell traffic: a summary of a National Institute of Child Health and Human Development-sponsored conference. J maternal-fetal neonatal medicine: Off J Eur Assoc Perinatal Medicine Fed Asia Oceania Perinatal Societies Int Soc Perinatal Obstet. (2003) 14:123–9. doi: 10.1080/jmf.14.2.123.129, PMID: 14629094

[51] Lo YM Lau TK Chan LY Leung TN Chang AM. Quantitative analysis of the bidirectional fetomaternal transfer of nucleated cells and plasma DNA. Clin Chem. (2000) 46:1301–9. doi: 10.1093/clinchem/46.9.1301, PMID: 10973858

[52] Dana M Fibach E. Fetal hemoglobin in the maternal circulation - contribution of fetal red blood cells. Hemoglobin. Mar. (2018) 42:138–40. doi: 10.1080/03630269.2018.1466712, PMID: 29745271

[53] Jørgensen J. Feto-maternal bleeding. During pregnancy at delivery. Acta obstetricia gynecologica Scandinavica. (1977) 56:487–90. doi: 10.3109/00016347709155017, PMID: 602720

[54] Sebring ES Polesky HF. Fetomaternal hemorrhage: incidence, risk factors, time of occurrence, and clinical effects. Transfusion. (1990) 30:344–57. doi: 10.1046/j.1537-2995.1990.30490273444.x, PMID: 2190367

[55] Dupre AR Morrison JC Martin JN Jr. Floyd RC Blake PG. Clinical application of the Kleihauer-Betke test. J Reprod Med. (1993) 38:621–4., PMID: 7692044

[56] Devi B Jennison RF Langley FA. Significance of placental pathology in transplacental haemorrhage. J Clin pathology. (1968) 21:322–31. doi: 10.1136/jcp.21.3.322, PMID: 4972435 PMC473790

[57] Scholz C Hermann C Kachler A Kainer F Friese K Makrigiannakis A. Association of placental inflammation with fetomaternal hemorrhage and loss of placental mucin-1. Arch gynecology obstetrics. (2012) 285:605–12. doi: 10.1007/s00404-011-2028-1, PMID: 21805141

[58] Kim YA Makar RS. Detection of fetomaternal hemorrhage. Am J hematology. (2012) 87:417–23. doi: 10.1002/ajh.22255, PMID: 22231030

[59] Christensen RD Lambert DK Baer VL Richards DS Bennett ST Ilstrup SJ. Severe neonatal anemia from fetomaternal hemorrhage: report from a multihospital health-care system. J perinatology: Off J California Perinatal Assoc. (2013) 33:429–34. doi: 10.1038/jp.2012.142, PMID: 23196720

[60] Laube DW Schauberger CW. Fetomaternal bleeding as a cause for "unexplained" fetal death. Obstet Gynecol. (1982) 60:649–51., PMID: 7145257

[61] Murphy KW Venkatraman N Stevens J. Limitations of ultrasound in the diagnosis of fetomaternal haemorrhage. BJOG: an Int J obstetrics gynaecology. (2000) 107:1317–9. doi: 10.1111/j.1471-0528.2000.tb11629.x, PMID: 11028590

[62] O'Leary BD Walsh CA Fitzgerald JM Downey P McAuliffe FM. The contribution of massive fetomaternal hemorrhage to antepartum stillbirth: a 25-year cross-sectional study. Acta obstetricia gynecologica Scandinavica. (2015) 94:1354–8. doi: 10.1111/aogs.12762, PMID: 26332994

[63] Ebbing C Rasmussen S Kiserud T. Middle cerebral artery blood flow velocities and pulsatility index and the cerebroplacental pulsatility ratio: longitudinal reference ranges and terms for serial measurements. Ultrasound obstetrics gynecology: Off J Int Soc Ultrasound Obstetrics Gynecology. (2007) 30:287–96. doi: 10.1002/uog.4088, PMID: 17721916

[64] Zimmerman R Carpenter RJ Jr. Durig P Mari G. Longitudinal measurement of peak systolic velocity in the fetal middle cerebral artery for monitoring pregnancies complicated by red cell alloimmunisation: a prospective multicentre trial with intention-to-treat. BJOG: an Int J obstetrics gynaecology. (2002) 109:746–52. doi: 10.1111/j.1471-0528.2002.01314.x, PMID: 12135209

[65] Oepkes D Seaward PG Vandenbussche FP Windrim R Kingdom J Beyene J. Doppler ultrasonography versus amniocentesis to predict fetal anemia. New Engl J Med. (2006) 355:156–64. doi: 10.1056/NEJMoa052855, PMID: 16837679

[66] Lees CC Romero R Stampalija T Dall'Asta A DeVore GA Prefumo F. Clinical Opinion: The diagnosis and management of suspected fetal growth restriction: an evidence-based approach. Am J obstetrics gynecology. (2022) 226:366–78. doi: 10.1016/j.ajog.2021.11.1357, PMID: 35026129 PMC9125563

[67] Shih IM Kurman RJ. Ki-67 labeling index in the differential diagnosis of exaggerated placental site, placental site trophoblastic tumor, and choriocarcinoma: a double immunohistochemical staining technique using Ki-67 and Mel-CAM antibodies. Hum pathology. (1998) 29:27–33. doi: 10.1016/s0046-8177(98)90386-0, PMID: 9445130

[68] Shih IM Kurman RJ. p63 expression is useful in the distinction of epithelioid trophoblastic and placental site trophoblastic tumors by profiling trophoblastic subpopulations. Am J Surg pathology. (2004) 28:1177–83. doi: 10.1097/01.pas.0000130325.66448.a1, PMID: 15316317

