# Supplementary material for: Intraplacental choriocarcinoma and spontaneous fetomaternal hemorrhage: Uncovering diagnostic clues in a challenging case
Source: Front Oncol. 2025 Sep 1;15:1600200. doi: 10.3389/fonc.2025.1600200 (PMC12433862; doi:10.3389/fonc.2025.1600200)
Supplement: Supplementary file 1 [file DataSheet1.docx]

Search: ((fetomaternal haemorrhage) OR (FMH)OR (fetomaternal transfusion)) AND ((choriocarcinoma) OR (intraplacental choriocarcinoma) OR (intraplacental choriocarcinoma) OR (Gestational Trophoblastic Disease) OR (Gestational Trophoblastic Neoplasia) OR (Gestational Trophoblastic Tumors) OR (Placental Trophoblastic tumor))

Sort by: Publication Date

("fetomaternal haemorrhage"[All Fields] OR "fetomaternal transfusion"[MeSH Terms] OR ("fetomaternal"[All Fields] AND "transfusion"[All Fields]) OR "fetomaternal transfusion"[All Fields] OR ("fetomaternal"[All Fields] AND "hemorrhage"[All Fields]) OR "fetomaternal hemorrhage"[All Fields] OR "FMH"[All Fields] OR ("fetomaternal transfusion"[MeSH Terms] OR ("fetomaternal"[All Fields] AND "transfusion"[All Fields]) OR "fetomaternal transfusion"[All Fields])) AND ("choriocarcinoma"[MeSH Terms] OR "choriocarcinoma"[All Fields] OR "choriocarcinomas"[All Fields] OR ("intraplacental"[All Fields] AND ("choriocarcinoma"[MeSH Terms] OR "choriocarcinoma"[All Fields] OR "choriocarcinomas"[All Fields])) OR ("intraplacental"[All Fields] AND ("choriocarcinoma"[MeSH Terms] OR "choriocarcinoma"[All Fields] OR "choriocarcinomas"[All Fields])) OR ("gestational trophoblastic disease"[MeSH Terms] OR ("gestational"[All Fields] AND "trophoblastic"[All Fields] AND "disease"[All Fields]) OR "gestational trophoblastic disease"[All Fields]) OR ("gestational trophoblastic disease"[MeSH Terms] OR ("gestational"[All Fields] AND "trophoblastic"[All Fields] AND "disease"[All Fields]) OR "gestational trophoblastic disease"[All Fields] OR ("gestational"[All Fields] AND "trophoblastic"[All Fields] AND "neoplasia"[All Fields]) OR "gestational trophoblastic neoplasia"[All Fields]) OR (("gestate"[All Fields] OR "gestated"[All Fields] OR "gestates"[All Fields] OR "gestating"[All Fields] OR "gestational"[All Fields] OR "gestations"[All Fields] OR "pregnancy"[MeSH Terms] OR "pregnancy"[All Fields] OR "gestation"[All Fields]) AND ("trophoblastic tumours"[All Fields] OR "trophoblastic neoplasms"[MeSH Terms] OR ("trophoblastic"[All Fields] AND "neoplasms"[All Fields]) OR "trophoblastic neoplasms"[All Fields] OR ("trophoblastic"[All Fields] AND "tumors"[All Fields]) OR "trophoblastic tumors"[All Fields])) OR ("trophoblastic tumor, placental site"[MeSH Terms] OR ("trophoblastic"[All Fields] AND "tumor"[All Fields] AND "placental"[All Fields] AND "site"[All Fields]) OR "placental site trophoblastic tumor"[All Fields] OR ("placental"[All Fields] AND "trophoblastic"[All Fields] AND "tumor"[All Fields]) OR "placental trophoblastic tumor"[All Fields]))

Translations

fetomaternal haemorrhage: "fetomaternal haemorrhage"[All Fields] OR "fetomaternal transfusion"[MeSH Terms] OR ("fetomaternal"[All Fields] AND "transfusion"[All Fields]) OR "fetomaternal transfusion"[All Fields] OR ("fetomaternal"[All Fields] AND "hemorrhage"[All Fields]) OR "fetomaternal hemorrhage"[All Fields]

fetomaternal transfusion: "fetomaternal transfusion"[MeSH Terms] OR ("fetomaternal"[All Fields] AND "transfusion"[All Fields]) OR "fetomaternal transfusion"[All Fields]

choriocarcinoma: "choriocarcinoma"[MeSH Terms] OR "choriocarcinoma"[All Fields] OR "choriocarcinomas"[All Fields]

choriocarcinoma: "choriocarcinoma"[MeSH Terms] OR "choriocarcinoma"[All Fields] OR "choriocarcinomas"[All Fields]

choriocarcinoma: "choriocarcinoma"[MeSH Terms] OR "choriocarcinoma"[All Fields] OR "choriocarcinomas"[All Fields]

Gestational Trophoblastic Disease: "gestational trophoblastic disease"[MeSH Terms] OR ("gestational"[All Fields] AND "trophoblastic"[All Fields] AND "disease"[All Fields]) OR "gestational trophoblastic disease"[All Fields]

Gestational Trophoblastic Neoplasia: "gestational trophoblastic disease"[MeSH Terms] OR ("gestational"[All Fields] AND "trophoblastic"[All Fields] AND "disease"[All Fields]) OR "gestational trophoblastic disease"[All Fields] OR ("gestational"[All Fields] AND "trophoblastic"[All Fields] AND "neoplasia"[All Fields]) OR "gestational trophoblastic neoplasia"[All Fields]

Gestational: "gestate"[All Fields] OR "gestated"[All Fields] OR "gestates"[All Fields] OR "gestating"[All Fields] OR "gestational"[All Fields] OR "gestations"[All Fields] OR "pregnancy"[MeSH Terms] OR "pregnancy"[All Fields] OR "gestation"[All Fields]

Trophoblastic Tumors: "trophoblastic tumours"[All Fields] OR "trophoblastic neoplasms"[MeSH Terms] OR ("trophoblastic"[All Fields] AND "neoplasms"[All Fields]) OR "trophoblastic neoplasms"[All Fields] OR ("trophoblastic"[All Fields] AND "tumors"[All Fields]) OR "trophoblastic tumors"[All Fields]

Placental Trophoblastic tumor: "placental trophoblastic tumour"[All Fields] OR "trophoblastic tumor, placental site"[MeSH Terms] OR ("trophoblastic"[All Fields] AND "tumor"[All Fields] AND "placental"[All Fields] AND "site"[All Fields]) OR "placental site trophoblastic tumor"[All Fields] OR ("placental"[All Fields] AND "trophoblastic"[All Fields] AND "tumor"[All Fields]) OR "placental trophoblastic tumor"[All Fields]
