# Supplementary figures and images for: Intraplacental choriocarcinoma and spontaneous fetomaternal hemorrhage: Uncovering diagnostic clues in a challenging case
Source: Front Oncol. 2025 Sep 1;15:1600200. doi: 10.3389/fonc.2025.1600200 (PMC12433862; doi:10.3389/fonc.2025.1600200)

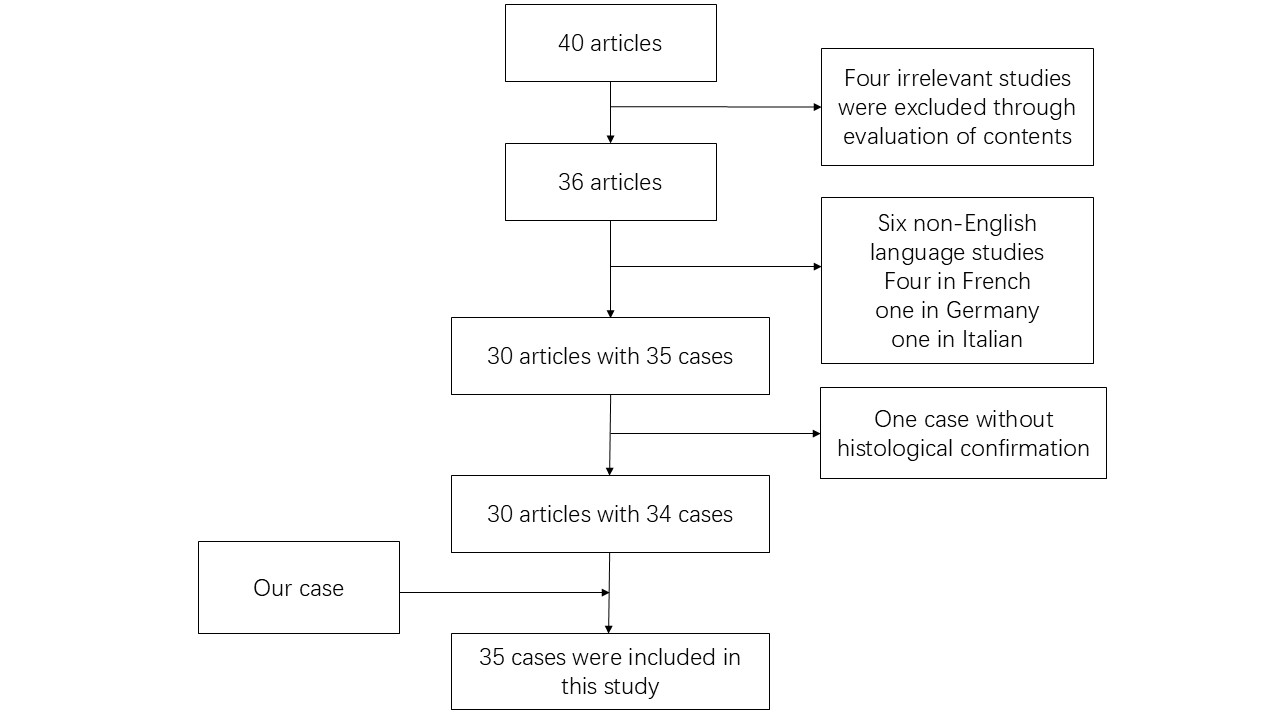

Supplement: Supplementary file 2 [file Image1.jpeg]
